# Role of the NLRP3 Inflammasome: Insights Into Cancer Hallmarks

**DOI:** 10.3389/fimmu.2020.610492

**Published:** 2021-02-03

**Authors:** Ting-Yi Lin, Meng-Chun Tsai, Wei Tu, Hsin-Chih Yeh, Shu-Chi Wang, Shu-Pin Huang, Chia-Yang Li

**Affiliations:** ^1^ School of Medicine, College of Medicine, Kaohsiung Medical University, Kaohsiung, Taiwan; ^2^ Department of Urology, Kaohsiung Medical University Hospital, Kaohsiung, Taiwan; ^3^ Department of Urology, Kaohsiung Municipal Ta-Tung Hospital, Kaohsiung, Taiwan; ^4^ Department of Urology, Faculty of Medicine, College of Medicine, Kaohsiung Medical University, Kaohsiung, Taiwan; ^5^ Department of Medical Laboratory Science and Biotechnology, Kaohsiung Medical University, Kaohsiung, Taiwan; ^6^ Graduate Institute of Medicine, College of Medicine, Kaohsiung Medical University, Kaohsiung, Taiwan; ^7^ Center for Cancer Research, Kaohsiung Medical University, Kaohsiung, Taiwan; ^8^ Department of Medical Research, Kaohsiung Medical University Hospital, Kaohsiung, Taiwan

**Keywords:** NLRP3 inflammasome, cancer hallmarks, tumor microenvironment, pyroptosis, interleukin-1β

## Abstract

In response to a variety of stresses, mammalian cells activate the inflammasome for targeted caspase-dependent pyroptosis. The research community has recently begun to deduce that the activation of inflammasome is instigated by several known oncogenic stresses and metabolic perturbations; nevertheless, the role of inflammasomes in the context of cancer biology is less understood. In manipulating the expression of inflammasome, researchers have found that NLRP3 serves as a deterministic player in conducting tumor fate decisions. Understanding the mechanistic underpinning of pro-tumorigenic and anti-tumorigenic pathways might elucidate novel therapeutic onco-targets, thereby providing new opportunities to manipulate inflammasome in augmenting the anti-tumorigenic activity to prevent tumor expansion and achieve metastatic control. Accordingly, this review aims to decode the complexity of NLRP3, whereby summarizing and clustering findings into cancer hallmarks and tissue contexts may expedite consensus and underscore the potential of the inflammasome in drug translation.

## Introduction

Cancer is a significant public health problem, and cancer deaths are rapidly increasing worldwide (1). In 2000, Drs. Douglas Hanahan and Robert A. Weinberg proposed six hallmarks of cancer, including sustaining proliferative signaling, evading growth suppressors, resisting cell death, enabling replicative immortality, inducing angiogenesis, and activating invasion and metastasis (2). Over the following decade, the authors summarized the latest discoveries into four more hallmarks upon discoveries, such as deregulating cellular energetics, avoiding immune destruction, tumor-promoting inflammation, and genome instability and mutation (3). These universal hallmarks are vital for maintaining tumor development, growth, and adaptation.

**Figure 1:**
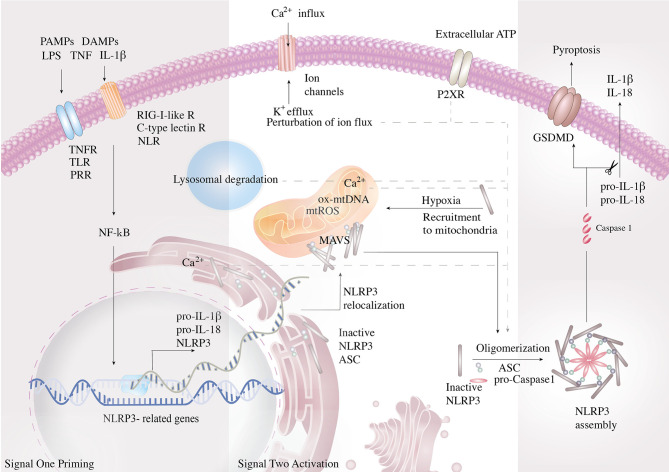
Mechanism and regulation of NLRP3 inflammasome activation. NLRP3 inflammasome is activated in a two-step process. First, NF-*κ*B is initially activated by the pathogen-associated molecular patterns (PAMPs)- and damage-associated molecular pattern (DAMP)-mediated signal cascades and induces the transcription of NLRP3 inflammasome components and the inactive un-spliced cytokines. PAMPs are recognized by pattern-recognition receptors (PRRs, including Toll-like receptors, NLRs, RIG-I-like receptors, C-type lectin receptors). Signal two assembles the inflammasome complex via oligomerization and activates caspase-1, the pore-forming protein gasdermin D (GSDMD), and the inflammatory cytokines. Signal two arises from lysosomal activity. Signal two activating DAMPs consist of perturbation of ion flux, extracellular adenosine triphosphate (ATP), lysosomal degradation, mitochondrial reactive oxygen species (mtROS), and oxidized mitochondrial DNA (ox-mtDNA). To improve efficiency of oligomerization, the organelles work in conjunction to strategically bring the components close to the vital organelles and then together. Resting NLRP3 localizes to endoplasmic reticulum, whereas it is redistributed to mitochondria via mitochondria-associated adaptor molecule (MAV) when activated.

Adding a layer of complexity to the oncogenic process, overlapping crosstalk between tumor microenvironment (TME) and local inflammation fuels oncogenic initiation, development and progression. Furthermore, disturbance of the cellular milieu generated by the byproducts of inflammation exacerbates genome instability that promotes the neoplastic disease’s plasticity to expedite tumorigenesis and precipitate multiple hallmarks. Generally poised as the activator of procaspase-1, inflammasomes are cytosolic multi-protein complexes that predominantly engage with innate immunity defense *via* activation of pro-interleukin (IL)-1β and pro-IL-18 cytokines (4). The active form of IL-18 is essential for interferon-gamma (IFN-**γ**) production, which augments the cytotoxicity potential of natural killer (NK) cells and T cells (5). Active caspase-1, the pore-forming protein gasdermin D (GSDMD) that disrupts the osmotic potential and results in pyroptosis, is a form of programmed cellular death (6). Inflammasome consists of a danger sensor (nucleotide-binding and oligomerization domain-like receptors, NLR), an adaptor (apoptosis-associated speck-like protein containing a CARD, ASC), and an effector protease (caspase-1) (7). Activation of NLRs with the assembly of the inflammasome requires two sequential activation signals to exert optimal effector response. The cell is initially primed by signal one with the activation of the tumor necrosis factor (TNF), IL-1β, or pathogen-associated molecular patterns (PAMPs) to enable the transcriptional upregulation of NLRP3 inflammasome components and the inactive unspliced cytokines. The inflammasome components are then oligomerized *via* signal two delivery, consisting of PAMPs or damage-associated molecular patterns (DAMPs). PAMPs are recognized by pattern-recognition receptors (PRRs, including Toll-like receptors, NLRs, RIG-I-like receptors, C-type lectin receptors, etc.), which play a key role in innate immunity for the recognition of pathogens or of cellular injury (8). The DAMPs responsible for NLRP3 activation include a myriad of processes such as perturbation of ion flux, extracellular adenosine triphosphate (ATP), lysosomal degradation, mitochondrial reactive oxygen species (mtROS), and oxidized mitochondrial DNA (ox-mtDNA) (9). The inflammasome cleaves the caspase1-dependent release of the pro-inflammatory cytokines IL-1β, IL-18, and activates pyroptosis (**Figure 1**) **(** 10).

The role of inflammasomes in the context of cancer biology is less understood. NLRP3 recognizes a diverse set of inflammation-inducing stimuli as an intracellular danger sensor, including PAMPs and DAMPs. During cancer expansion, metabolites are actively secreted to the TME as extracellular messengers. ATP, one of the major cancer metabolites and constituents of the TME (11, 12), serves as a key DAMP that activates NLRP3 inflammasome *via* the purinergic P2X7 receptors (13, 14). The P2XR mediates host–tumor cell interactions and triggers NLRP3 activation *via* cytosolic Ca^2+^ influx and K^+^ efflux, thereby shaping the TME and tumor fate. Besides, hypoxia has been demonstrated to prime the activation of NLRP3 inflammasome (15, 16). Since the TME possesses hypoxic and inflamed sites that accumulate with extracellular ATP, the impacts of NLRP3 inflammasome on the progression of tumor and maintenance of TME have become a critical issue. Herein, we discuss the emerging literature and highlight the role of NLRP3 in oncogenic development by dissecting the functions and justifying each cancer hallmark to gain a comprehensive understanding.

## Role of NLRP3 in Regulating the Hallmarks of Cancer

### Tumor-Promoting Inflammation

Inflammation is frequently observed at the earliest stage of neoplastic initiation and propels oncogenic development in several types of cancer (17). As a danger-sensing protein complex, NLRP3 is activated by numerous oncogenic inducers such as *Helicobacter pylori* (*H. pylori*), human papillomavirus (HPV), uric acid, crystals, and reactive oxygen species (ROS) (18). *H. pylori* infection enhances NLRP3 expression in gastric cancer and triggers the uncontrolled proliferation of epithelial cells (18). Similarly, inflammasome activation in prostate tissue is mediated by uric acid, crystals, and infections increase the risks of prostate gland injury and prostate cancer progression (19). NLRP3-mediated tumorigenesis is observed not only through its downstream activations, but in the upstream root of genetic variants as well. Pontillo et al. discovered that a variant in the *NLRP3* gene, *rs10754558* (20) was associated with HPV resistance and showed a statistically significant relationship between *rs10754558* and cervical cancer development (21). Epistasis analysis revealed that *NLRP3* variants together with polymorphisms in inflammasome-related genes modulate both the frequency of inflammasome activation and the process of IL-1β and IL-18 maturation that influence HPV infection outcome and cervical cancer progression (20) (**Table 1**).

**Table 1:** Inflammation-associated cancer hallmarks.

Cancer type	Mechanism	Reference
***Tumor-promoting inflammation***		
Cervical	NLRP3 is implicated in numerous oncogenic stress such as *H. pylori*, HPV	(18)
Cervical	NLRP3 variants together with polymorphisms influence progression outcome	(20)
Prostate	Pathogens are a stimulatory factor for NLRP3 activation in prostate cancer	(21)
***Evading immune destruction***		
Breast	NLRP3 upregulates sphingosine-1-phosphate (S1P) signaling in TAM formation	(22)
Breast	IL-1β suppresses T cell proliferation	(23)
Breast	IL-1β promotes metastasis to bone and lung *via* the recruitment of *γ/δ* T cells.	(23)
CRC, liver metastasis	Blocking NLRP3 signaling suppresses tumor cell migration	(24)
Fibrosarcoma, lymphoma	Absence of NLRP3 abolishes the immunosurveillance against hyperploid cells	(25)
Multiple cancers	Activation of NLRP3 in TAMs promotes metastatic spread	(22–26)
Pancreas	NLRP3 promotes survival *via* NF-*κ*B signaling and limited cytotoxic immune cell infiltration	(27)
Pancreas	PDL1 and CTLA4 regulates NLRP3 activation	(28)
Skin	Inhibition of the NLRP3 in TAM suppresses metastasis	(26)
Skin, bone metastasis	NLRP3 suppresses NK and T cell-mediated anti-tumor actions and promotes tumor cell survival	(29)
***Enforcing immune surveillance***		
CRC, liver metastasis	NLRP3 suppresses hepatic metastasis of CRC by promoting NK cytotoxic ability	(30)
HNSCC	NLRP3 reshapes the anti-tumor response through reducing immunosuppressive cells	(31)
NPC	NLRP3 increases IL-1β that inhibits tumor growth and prevents local relapse by recruiting antitumor N1 tumor-associated neutrophils	(32)

### Evading Immune Destruction

Immunogenic reprogramming of innate and adaptive immunity within the TME has brought to light the importance of NLRP3 signaling in primary tumors or innate immune cells in mediating immune evasion that not only promotes cancer dissemination but also allows clonogenic survival. Here we discuss the role of NLRP3 activation in immune cells and primary tumor as a failsafe mechanism in inflammation-mediated immune surveillance. Highly immunogenic cancer cells evade immune surveillance by disabling the immune-components or downregulating immunogenic antigens. Recent progress in the action of NLRP3 witnessed remarkable adeptness in paralyzing immune cells dispatched to eliminate cancer by immunosuppressive secretions. The robust immune evasion was emphasized when Aranda et al. demonstrated that the absence of NLRP3 partially abolished immune surveillance against hyperploid tumors (25). Due to the inherent immunogenicity of extra chromosomes, hyperploid cells rarely form tumors or are compromised into slowly growing tumors *in vivo* (25, 33) (**Table 1**).

The abundance of immune regulatory subsets of tumor-infiltrating leukocytes in the TME is correlated with disease progression and poor prognosis (34) such as M2 macrophages, myeloid-derived suppressor cells (MDSC), neutrophil subsets, regulatory dendritic cells (DC), and regulatory T cells (Tregs) (35, 36). In sarcoma and metastatic melanoma models, NLRP3 activation enhances MDSC and Treg populations while suppressing both NK and T cell-mediated tumor surveillance. Zhiyu et al. found that *NLRP3* silencing resulted in a five-fold reduction of MDSCs in host mice (29). These NLRP3-deficient mice were also presented with less pulmonary metastasis in an orthotopic transplant mouse model of mammary adenocarcinoma (29, 37). NLRP3 expression in tumor-infiltrating macrophages is correlated with survival, lymph node invasion, and metastasis of mammary carcinoma patients (22) (**Table 1**).

Among tumor-infiltrating immune cells, tumor-associated macrophages (TAMs) take center stage in promoting both metastatic tumor spread and immune evasion (22, 24, 26). NLRP3 mediated macrophage-colorectal cancer (CRC) cell crosstalk and achieved higher migration rates of CRC cells, whereas blocking NLRP3 signaling suppressed CRC cell migration *in vitro* and liver metastatic ability *in vivo* (24). In NLRP3- or caspase-1-knockout macrophages, the ability to promote the migration and invasion of melanoma cells was, similarly, greatly diminished and the metastatic potential of melanoma tumor cells was suppressed (26). Mediating immune-suppressive crosstalk, NLRP3 signaling in macrophages has been reported to decrease NK cell activation (21), reduce Th1 cell polarization (27), and attenuate cytotoxic CD8^+^ T cell activation (27). The suppression of anti-tumor immune responses is accompanied by enhanced differentiation of CD4^+^ T cells into tumor-promoting cells such as T helper type 2 cells (Th2 cells), Th17 cells, and Treg populations (27). NLRP3 also plays a role in treatment response where chemotherapeutic agents were shown to activate NLRP3 activation in MDSC, leading to immunosuppressive responses through IL-1β production that blunts the anti-tumor therapeutic effect (29, 38) (**Table 1**).

IL-1β is abundant in the TME, where this cytokine can promote tumor growth and presents anti-tumor activities; thus, alteration of TME from pro-invasive inflammation towards anti-tumor cell immunity against an overwhelming immunosuppressive situation is a strategy for treating cancer (35). Breast cancer-associated fibroblast (CAF)-derived IL-1β in TME drives an immunosuppressive phenotype by promoting TAM’s reprogramming to an M2-like phenotype and restricting the accumulation of T cells in tumors (23). Daley et al. found that inhibition of NLRP3, ASC or caspase-1 protects against pancreatic ductal adenocarcinoma (PDA) *via* immunogenic reprogramming of innate and adaptive immunity within the TME (27). NLRP3 inflammasome activation in tumor cells can endorse a chronic inflammatory TME that encourages malignant transformation and extinguishes the local immunity provided by NK or T cells (31, 39). IL-1β, has also been reported to recruit and activate *γ/δ* T cells involved in mediating breast cancer metastasis to bone and lung metastasis of melanoma and breast cancer (23) (**Table 1**). Not only does the cytokine dampen immune surveillance, but it also blunts the anti-tumor therapeutic effect of chemotherapeutic agents *via* drug-induced NLRP3 activation in MDSC as well (29, 38) (**Table 1**).

Immune checkpoint inhibitors targeting cytotoxic T-lymphocyte-associated protein 4 (CTLA4) and programmed cell death protein 1 (PD1) and its ligand-programmed death ligand 1 (PDL1) axis appear to require a pre-existing, primed effector anti-tumor CD8^+^ T cell infiltration for optimum therapeutic effect (28, 40). Among the known human chemokines, a co-regulated set of four (chemokine (C-C motif) ligand (CCL)-4, CCL-5, chemokine (C-X-C motif) ligand (CXCL)-9, CXCL-10) chemokines is upregulated in primary PDA carcinoma and PDA liver metastasis, which regulates CD8^+^ T cell infiltration, activates T cells, and promotes NLRP3-mediated T cell priming and enhances anti-tumor CD8^+^ T cell cytotoxic activity for an effective immune checkpoint therapy response. (**Table 1**). To reiterate the role of NLRP3 in enhancing immune checkpoint inhibitors, Kaplanov et al. found that although anti-PD-1 reduced tumor growth, the combination of anti-IL-1β plus anti-PD-1 abrogated the tumors completely. These observations support the findings of clinical trials that blockage of IL-1β in cancer might serve as a checkpoint inhibitor (35).

Despite accumulating evidence supporting NLRP3-mediated immune evasion, the inflammasome has also been reported to activate pro-surveillance pathways that heightened anti-tumor control and prevented tumor metastasis. The innate immune system may sense danger signals by the activation of the NLRP3 inflammasome, where downstream crosstalk engages NK cell cytolytic activity, the primarily cytotoxic cell regulating metastatic control. NLRP3 in the primary lesion of cancer cells drives the production of pro-IL-1β, DC maturation, and the secretion of IL-1β to support the evolution of tumor-specific CD8^+^ T cells (41). Moreover, Dupaul-Chicoine et al. found that the activation of NK cells was IL-18 dependent, but IFN-*γ* independent. The inflammasome-mediated tumor suppression is proposed to be primed by IL-18, promoting hepatic NK cell maturation, and aims to target FasL-sensitive tumor cells (30). IL-18 also plays an important role in regulating lymphocyte chemoattractant properties and promoting the expression of pro-inflammatory mediators, angiogenesis and adhesion-related factors (42–44). In a separate study, Dagenais et al. found that IL-18-deficient mice reveal a higher metastatic burden than wild-type mice in CRC liver metastasis, suggesting that IL-18 plays an important role in CRC metastatic growth restriction (45). Besides, in an animal model lacking T, B, and NKT cells, Rag1^−/−^ mice exhibit a similar tumor metastatic burden compared to wild-type mice; however, Rag1^−/−^/IL-18^−/−^ mice presented with an increased metastatic burden, indicating that IL-18-induced tumor cytotoxicity is independent of the major adaptive immunity effectors such as cytotoxic T lymphocytes (45).

In the primary lesion of cancer cells, NLRP3 drives the production of pro-IL-1β, DC maturation, and the secretion of IL-1β to support the differentiation of tumor-specific CD8^+^ T cells (41). NLRP3 inflammasome has also been reported, in a head and neck squamous carcinoma (HNSC) mice model, to inhibit and delay tumor growth and reshape the anti-tumor response through a decrease in the number of immunosuppressive cells and an enhancement in the function of effector T cells (31). Not only does cancer NLRP3 play a role in adaptive immunity in enforcing immune surveillance, but it also mediates anti-tumor immunity in innate immunity through activation of tumor-associated neutrophils (TAN). Chen et al. observed that irradiated nasopharyngeal carcinoma (NPC) presented with increased NLRP3 signaling and higher levels of tumor-derived IL-1β, and proposed that these cytokines can inhibit tumor growth and prevent local relapse by recruiting antitumor N1 TAN. Correlating with the clinical scenario, Chen et al. found that the presence of TAN significantly promoted survival in NPC patients (32) (**Table 1**).

### Sustaining Proliferative Signaling

The mitogenic signaling in cancer cells is chronically sustained and undamped. It has been proposed that cancer cells achieve such a feat through various approaches: tumor cells can produce growth factor ligands independently, promote receptor’s structural alterations that facilitate ligand-independent activation, and elevate the levels of receptor proteins displayed at the surface resulting in the hyperresponsive reaction under the physiologic growth environment. The current literature has not reported on receptor alterations or dysregulation; nevertheless, NLRP3 has been associated with sending signals to stimulate normal cells within the tumor-associated stroma and supply them with growth factors. The crosstalk results with activation of the downstream constitutive pathway in modulating proliferation and cell cycle and dampening negative feedback: the regular operation to contain uncontrolled proliferation.

NLRP3 downstream IL-1β regulates diverse proliferative processes associated with inflammatory response (21, 42), cell cycle modulation (21, 46), nuclear factor *κ*B (NF-*k*B)-c-Jun N-terminal kinase (JNK) signaling (21) as well as colony survival (47). The critical role of IL-1β is brought to light in melanoma cells, as the late-stage human melanoma actively secretes IL-1β without exogenous stimulation (48, 49). The spontaneous secretion of IL-1β shows similar characteristics as auto-inflammatory diseases caused by gain-of-function mutations in NLRP3 (48, 49), suggesting that the constitutive secretion of IL-1β may play a role in the underlying pathogenesis of melanoma (48, 49). Another study exploring melanoma found that while late-stage melanoma expresses IL-1β constitutively, intermediate-stage melanoma requires activation of the IL-1 receptor to secrete active IL-1β, and early-stage melanoma requires stimulation of the IL-1 receptor plus the co-stimulant muramyl dipeptide to secrete active IL-1β. These findings reinforce that IL-1-mediated autoinflammation paves the way to human melanoma development and progression (49). In breast cancer, numerous studies have reported a pro-tumorigenic role of IL-1β, whereas blocking IL-1β reduces tumor growth (50–52). Moreover, Voigt et al. indicated a previously unrecognized mechanism by which cancer cells induce IL-22 production from memory CD4^+^ T cells *via* activation of the NLRP3 inflammasome in breast cancer. The authors also demonstrated that the IL-1 receptor antagonist anakinra abrogates IL-22 production and reduces tumor growth in a murine breast cancer model (50) (**Table 2**).

**Table 2:** Proliferation- and cell death-associated cancer hallmarks.

Cancer type	Mechanism	Reference
***Sustaining proliferative signaling***		
Breast	Reduced NLRP3 activation reduced angiogenesis, decrease proliferation and increase apoptosis	(53)
Breast	Inactivation of NLRP3 by miR-233 reduced proliferation	(54)
Breast	NLRP3 and release of IL-1β promote tumor growth	(50, 51)
Breast	NLRP3 by leptin plays a critical role in the growth of breast cancer cells *via* suppression of cell cycle	(46)
Colon	Activation of NLRP3 inflammasomes promotes tumor development	(42)
Endometrial	Activation of NLRP3 promotes cancer progression	(55)
Gastric	NLRP3 increases cell differentiation by engaging cyclin-D1 and inducing IL-1β production that activates NF-*κ*B/JNK signaling to cause cell proliferation	(21)
Gastric	NLRP3 triggers uncontrolled proliferation of epithelial cells and tumorigenesis	(18)
Glioma	NLRP3 downregulation significantly inhibited the proliferation	(56)
HCC	Increased NLRP3 inflammasome represses proliferation and metastasis	(21)
HNSCC	Inhibition of NLRP3 inflammasome delayed tumor growth	(31)
Leukemia	Increased NLRP3 expression inhibits cell proliferation and stimulates apoptosis	(57)
Lung	NLRP3 leads to inflammatory response and promotes cancer initiation and progression	(21)
Lung	Inactivation of NLRP3 increases proliferation *via* activation of the NF-*κ*B signaling pathway	(54)
Melanoma	Reduced NLRP3 and IL-1β expression inhibited carcinogenesis	(21)
Melanoma	Activation of NLRP3 increases IL-1β, regulates tumor aggression	(48)
Melanoma	Activation of NLRP3, increases IL-1β, promotes proliferation and angiogenesis in melanoma	(49)
OSCC	Decreased NLRP3 significantly decreased the cell viabilities and affected the colony formation of OSCC cells	(47)
Ovarian	Inactivation of NLRP3 by miR-233 regulated ovarian cancer cell proliferation	(54)
***Evading growth suppressors***		
Breast	NLRP3 suppresses apoptosis and progression of cell cycle	(46)
Breast	Inhibition of NLRP3 induces cell cycle arrest at the G0/G1 phase	(58)
Gastric	NLRP3 promotes its transcription in epithelial cells *via* binding CCND1 promoter	(18)
***Enabling Replicative Immortality***		
Breast	NLRP3 agonists activate Wnt/*β*-catenin	(59)
Breast	NLRP3 serves as a driver of treatment resistance in cancer stem cells	(60)
Colon	ERs regulate NLRP3 *via* Wnt/*β*-catenin signaling pathway	(61)
Colon	IL-1β promotes stem cell development	(62)
Colon	IL-1β displays an up-regulation of stemness factor genes (Bmi1 and Nestin) and increases drug resistance, hallmarks of CSCs	(63)
HNSCC	NLRP3 is associated with cancer stem cell markers BMI1, ALDH1 and CD44	(62)
Intestine	IL-1β enhances expression of Bmi-1, Lgr-5, c-Myc, Nanog, and *β*-catenin	(64)
Melanoma	IL‐1β increases stem cell ability to self‐renew, possess marker expression and invasive capacity	(65)
***Resisting cell death***		
Breast	Inactivation of NLRP3 inhibits suppression of apoptosis	(46)
Breast	NRLP3 and the production of pro-IL1β support differentiation of CD8^+^ T cells	(41)
Breast	NLRP3 inflammasome inactivation, driven by miR-223-3p inhibits apoptosis	(54)
Breast	Reduced NLRP3 activation increases apoptosis	(53)
Colon	IL-18 signaling controls colon tissue regeneration and re-epithelialization	(42)
Colon	IL-1β and IL-18 deficient mice enhance tumorigenesis	(66)
Glioma	si-NLRP3 reduced cell apoptosis rates	(56)
HCC	NLRP3 are down-regulated in hepatic parenchymal cells derived from liver cancer	(67)
Lung	Decreased NLRP3 inhibits apoptosis and is correlated with overexpression of miR-233.	(54)
Lung	Activation of NLRP3 transmits the signal in GSDMD-deficient tumor cells induced apoptosis	(68)
Lung	NLRP3 increases proliferation and migration by releasing IL-1β and IL-18	(69)

Varying levels of IL-1β operate distinct cellular signaling pathways and cell fate. Subjected to high levels of IL-1β, cells engage apoptotic pathways, whereas moderate levels of IL-1β peptides stimulate clonal expansion. Roy et al. generated stable IL-1β overexpressed MCF7 cells. In secreting high levels of IL-1β clone, cells underwent apoptosis due to genotoxic stress, whereas moderate IL-1β expressed cells undergoing clonal expansion. These studies support that concentrations of the IL-1β secretion determine its stimulatory or inhibitory signals regulating the growth of tumors that might result in novel preventive strategies (70). NLRP3-mediated IL-1β production activates NF-*κ*B and initiates JNK signaling to cause proliferation in gastric cancer and CRC (21, 39, 71). Similarly, activation of NLRP3 inflammasome in mesothelial cells of lung cancer leads to an inflammatory response that fuels cancer initiation and progression (21) and then activates the NF-*κ*B-signaling pathway in lung cancer, consequently increasing proliferation and inhibiting apoptosis (54). On the other hand, Salaro et al. found that NLRP3 overexpression inhibits cell proliferation and stimulates apoptosis in leukemic cells. We suggest the opposite results in NLRP3-mediated cell proliferation due to different IL-1β levels (57) (**Table 2**).

Manipulation of NLRP3 downstream components holds potential in determining cell fate. A central component of the inflammasomes, ASC, has been shown to promote tumor development favoring inflammation in infiltrating immune cells (42). Inactivation of NLRP3 inflammasome driven by miR-233-3p has been found to decrease the expression of NLRP3 inflammasome-associated proteins, ASC, IL-1β, and IL-18 in breast cancers and suppress tumor growth. In addition, inactivation of NLRP3 inflammasome has also been found to reduce IL-1β expression and halt development of melanoma (21). Yin et al. found the expression levels of NLRP3, ASC, caspase-1, and IL-1β are highly expressed in high-grade glioma compared to low-grade glioma, indicating an important role for NLRP3 in glioma malignancy. Overexpression of NLRP3 promotes cell proliferation and colony formation in glioma cells, whereas the downregulation of NLRP3 significantly repressed colony formation in glioma cells (56).

Similarly, NLRP3 expression levels are also correlated with the tumor size, lymph node metastatic status and IL-1β expression in oral squamous cell carcinoma (OSCC), and downregulating NLRP3 expression markedly attenuates the proliferation, migration, and invasion of OSCC (47). These results suggest that NLRP3 exerts a vital role in cancer cell proliferation. NLRP3 inflammasomes mediate both suppressions of apoptosis and progression of the cell cycle by leptin-dependent ROS production in breast cancer, which is mediated *via* estrogen receptor alpha (ER*α*)/reduced nicotinamide adenine dinucleotide phosphate (NADPH) oxidase signaling (46). Moreover, NLRP3 downstream, IL-1β, also stimulates the production of ROS that, in turn, induces DNA damage and cancer development in CRC (42) (**Table 2**).

### Evading Growth Suppressors

Sustained proliferation despite the accumulation of endogenous stress and unresolvable metabolic dysregulations requires the cancer cells to circumvent programs that negatively regulate uncontrolled expansion. The replicative machinery intrinsically possesses numerous gatekeepers to maintain homeostasis, and these housekeeping genes integrate diverse signaling inputs to determine if the cell’s condition is suboptimal or normalized for subsequent cell-cycle progression. Previous studies have explored the role of inflammasome activation in the cell cycle mediated by leptin, and the authors found that leptin, a known risk factor for breast cancer, induces cyclin D1 expression (18, 58) and mediates cell cycle progression.

In contrast, physiologic growth becomes abolished when treating with NLRP3 inhibitor or knockout of NLRP3. The decreased population of cells in S- and G2-M phases post-NLRP3 inhibition demonstrates that the activation of NLRP3 inflammasome plays a critical role in leptin-induced cell cycle progression (46). Inflammasome inhibitions were also found to induce cell cycle arrest at the G0/G1 phase and elevated expression levels of negative cell cycle modulators, including p27^Kip^ and p53 (58). Interestingly, activation of NLRP3 inflammasome was only activated during cell cycle interphases by NEK7, mitosis associated with kinase. Known for its role in cellular division, NEK7 mediates centrosome duplication, DNA repair and mitotic spindle activity (72); nevertheless, NEK7 also mediates mitochondrial regulation, and expressions of the NLRP3 inflammasome have been observed. Liu et al. also found that NEK7 is involved in many NLRP3-associated diseases (72). In investigating NEK7’s dual function in regulating cell division and intracellular danger sensor, Shi et al. found that NLRP3 activation requires NEK7 and suggested that NEK7 serves as a cellular switch enforcing exclusive cell fate between inflammasome response and cell division (73) (**Table 2**).

### Enabling Replicative Immortality

A limited number of successive cell division cycles hard-wired into most normal cell lineages safeguard against uncontrolled proliferation. The natural barrier to proliferation, replicative senescence, is the cell’s innate protective measure against oncogenic progression where cells subjected to irreparable stress and uncontrolled growth are forced to undergo cell cycle arrest. Dong et al. found that NLRP3 inhibits senescence and enables replicative immortality through regulating the Wnt/*β*-catenin pathway *via* the thioredoxin-interacting protein (TXNIP)/NLRP3 axis (74). A Wnt/*β*-catenin pathway and NLRP3 was proposed by Xu et al. to be co-regulated by miR-20b, a miRNA targeting TXNIP. TXNIP knockdown or targeting by miR-20b resulted in a pro-tumorigenic phenotype with increased cell proliferation, inhibited cell senescence-reduced cell cycle modulators (p16 and p21), and decreased NLRP3 inflammasome-associated proteins (NLRP3 and cleaved caspase-1). These findings reveal that senescence antagonist miR-20b or TXNIP knockdown resulted in attenuated Wnt/*β*-catenin pathway *via* dysregulation of the TXNIP/NLRP3 axis (74) (**Table 2**).

Wnt signaling is implicated in a multitude of processes such as embryogenesis, carcinogenesis and stem cell pluripotency. In the realm of replicative mortality, Park et al. discovered a novel telomere-independent function of telomerase reverse transcriptase (TERT)/telomerase to amplify signaling by the Wnt pathway. The amplification is achieved by TERT serving as a cofactor of the *β*-catenin/lymphoid enhancer factor (LEF) transcription factor complex (75). Huang et al. reported that *β*-catenin promotes NLRP3 inflammasome activation, and silencing of β-catenin impairs NLRP3 activation (76). Nevertheless, Zheng et al. found a reciprocal relationship between NLRP3 and Wnt signaling. NLRP3 agonist induces Wnt/*β*-catenin activation, whereas inactivation of Wnt/*β*-catenin results in the inhibition of NLRP3, IL-1β. The authors propose that NLRP3 activation might enhance resistance to gemcitabine *via* IL-1*β*/epithelial–mesenchymal transition (EMT)/Wnt/*β*-catenin signaling (59). Attempting to explore the underlying pathway in colon cancer, Liu et al. indicated that estrogen receptors regulate the Wnt/*β*-catenin pathway through targeting NLR, and by comparing gene expression between normal and colon cancer tissues, they pointed out that Wnt/*β*-catenin pathway-associated genes are significantly differentially expressed in ER^−/−^ mice. In addition, selective estrogen receptorα antagonists promote *β*-catenin degradation *via* inhibition of NLRP3. Collectively, these results indicate that NLRs are potential cancer markers that mediate estrogen receptor/Wnt/*β*-catenin signaling axis (61) (**Table 2**).

Telomeres protect ends of chromosomes and govern the capability of cells to counteract progressive telomere erosions caused by cell divisions. Telomerase elongates specific telomere repeats to the ends of chromosomes, which is absent in non-immortalized cells but overexpressed in immortalized cells and cancer lineages. Employing late generation telomerase knockout mice, Kang et al. found that telomere dysfunction greatly impacts innate immune cells where they present with exaggerated lung inflammation and then increase mortality upon respiratory infection. In investigating the mechanism of dysregulation, telomere dysfunction elicits macrophage mitochondrial abnormality and triggers hyperactivation of the NLRP3 inflammasome. Moreover, Kang et al. demonstrated that the peroxisome proliferator-activated receptor-gamma coactivator 1-alpha (PGC-1*α*)/estrogen-related receptor alpha (ERR*α*) axis, controlled by the telomere status, is required not only for maintaining mitochondrial homeostasis but additionally for retaining the TNF-actin interacting protein 3 (AIP3)-dependent machinery during inflammasome activation (56) (**Table 2**). These results bring to light a previous unexplored crosstalk network between replicative immortality and inflammasome activation.

A previous study indicated that IL-1β stimulates the activation of hypoxic signaling pathways in cancer cells that are critical for regulating and maintaining the characteristics of cancer stem cells (CSCs), including undifferentiated phenotype, self-renewal capacity, and immortality (77). The available evidence suggests that the same sets of transcription factors are implicated in regulating distinct cell fates. These overlapping sets of target genes from developmental genetics and oncogenesis highlight the importance of CSCs in NLRP3-associated carcinogenesis. Inflammasomal production of IL-1β reinforces the cancer stem cell phenotype and contributes to its invasive capacity and survival resiliency (60, 62–65). Huang et al. found that CSC markers are significantly upregulated with enriched CSC population in an IL-1β treated CRC cell line and spherical human primary CRC, and these markers, such as BMI1, nestin, Nanog, and *β*-catenin, can be used for stemness evaluation. Consistently, Huang et al. demonstrated that cancer expression of stem cell markers BMI1, aldehyde dehydrogenase 1 (ALDH1) and CD44 are associated with NLRP3, and they proposed that inflammasome may regulate CSC (62) (**Table 2**). 

### Resisting Cell Death

Programmed cell death by apoptosis serves as a natural barrier against oncogenic development. Despite the major downstream event of NLRP3 inflammation formation of caspase-1 mediated pyroptosis, NLRP3 seems to mediate the dual-function of apoptosis and survival. Herein, elucidation of the NLRP3 signaling circuitry governing the apoptotic program may serve as the identification of the target gene for cell fate determination. When comparing liver cancer biopsies with non-cancerous samples, Wei et al. found that both mRNA and protein levels of NLRP3 are markedly down-regulated in hepatic parenchymal cells derived from liver cancer, suggesting an antitumorigenic role of NLRP3 hepatocellular carcinoma (67). NLRP3 has been associated with suppressing malignant progression by triggering the mitochondrial apoptotic pathway, enhancing immune-cytokine levels in the tumor microenvironment (39), and activating tumor-suppressive protein p53 in epithelial cells (42, 46, 78) (**Table 2**).

The apoptotic trigger is controlled by counterbalancing pro- and anti-apoptotic commands. Direct inhibition of NLRP3 or its downstream molecules is associated with oncogenic progression and anti-apoptotic effect. NLRP3 inflammasome inactivation, driven by miR-223-3p, increases proliferation, promotes invasion and inhibits apoptosis in breast cancer cells (54). *MyD88* knockout mice have been found defective in downstream IL-1β and IL-18 productions, and their downstream signaling reveals an increase in colonic epithelial proliferation and CRC tumorigenesis (66). Furthermore, NLRP3 activation has been proposed to mediate multiple pathways other than that of the caspase-1. NLRP3 inflammasome activation-induced IL-1β and IL-18 in lung cancer may work through mechanisms other than the caspase-1 pathway, indicating that NLRP3 inflammasome can mediate the release of IL-1β and IL-18 through caspase-1-dependent or -independent pathways (69). Moreover, in A549 lung cancer cells, NLRP3 has been demonstrated to associate with sending signals to stimulate normal cells within the tumor-associated stroma, supply them with growth factors, and regulate cell proliferation and migration by releasing IL-1β and IL-18 through an autocrine or paracrine manner (69). Although caspase-1 activation is the major downstream event of NLRP3 inflammasome assembly, recent studies have reported that NLRP3 inflammasome could also be activated by caspase-8 (79). Besides, caspase-8 is also involved in the maturation of IL-1β and IL-18 by inducing a non-canonical process of releasing IL-1β (80) and IL-18 (69, 81) (**Table 2**).

The attenuation of the NLRP3 downstream pyroptosis pathway promotes apoptosis (54, 68). When attempting to activate pyroptotic signaling in GSDMD-deficient NSCLC, another type of programmed cell death other than pyroptosis was activated. Gao et al. found that in GSDMD-deficient tumor cells, the depletion of GSDMD activated cleavage of caspase-3 and PARP that guided the cell fate towards the intrinsic mitochondrial apoptotic pathway. Furthermore, knockdown of GSDMD attenuates tumor proliferation by promoting apoptosis and inhibiting epidermal growth factor receptor (EGFR)/Akt signaling axis (68). On the other hand, the increase of NLRP3, ASC, caspase-1, and IL-1β proteins in human glioma tissues is significantly correlated with higher World Health Organization grades. Inhibition of NLRP3 suppresses the proliferation, migration and invasion, and promotes apoptosis in glioma cells, while in contrast, increased expression of NLRP3 significantly enhances the proliferation, migration and invasion as well as attenuating apoptosis in glioma cells (56) (**Table 2**). However, as to how damaged cells decide whether to continue living in a dysfunctional state or die, and how cancer cells escape the inflammasome-induced pyroptosis both remain unclear.

### Deregulating Cellular Energetics-Aerobic Glycolysis

Despite its inferiority in ATP production, glycolysis allows diversion of its metabolic products into rewarding intermediates for multiple biosynthetic pathways required for active cellular expansion. An altered metabolic environment and its metabolites provide both the priming signal to induce NLRP3 inflammasome transcription and the trigger signal for inflammasome formation/activation (82). He et al. pointed out that NLRP3 is overexpressed in non-small cell lung cancer (NSCLC) tumor tissues, while in contrast, depletion of NLRP3 reduces cell growth *in vitro* and *in vivo* as well as decreases glycolysis while enhancing mitochondrial respiration, where pathways contributing to ATP production and intermediate substrates metabolism serve as the Warburg effect switch (83). Consistently in NPC, Epstein–Barr virus latent membrane protein 1 (LMP1) promotes myeloid-derived suppressor cells MDSC expansion that promotes immune escape in the tumor microenvironment. Cai et al. found that LMP1 promotes the expression of multiple glycolytic genes, including glucose transporter 1 (GLUT1), and upregulates extra-mitochondrial glycolysis pathways in malignant cells. This metabolic reprogramming results in increasing expression of the NLRP3 inflammasome and its downstream effectors, including IL-1β, IL-6, and GM-CSF (84).

In further exploring the underlying mechanism, Moon et al. indicated that activation of the glycolytic phenotypes is intrinsically linked to NLRP3 inflammation; *e.g.*, inhibition of mTOR complex 1 (mTORC1) suppresses NLRP3 inflammasome activation, and its regulatory associated protein of mTOR (Raptor)/mTORC1 downstream HK1-dependent glycolysis is critical for NLRP3 inflammasome activation (85) (**Table 3**). Previous studies indicated that resting NLRP3 localizes to endoplasmic reticulum structures (89), whereas on inflammasome activation, both NLRP3 and its adaptors redistribute and co-localize with endoplasmic reticulum and mitochondria organelle clusters (90). Mitochondria-associated adaptor molecule (MAVS) is required for optimal NLRP3 inflammasome activity including mediating recruitment of NLRP3 to mitochondria, promoting production of IL-1β, and modulating the pathophysiologic activity of the NLRP3 inflammasome *in vivo* (91). In addition, Kim et al. found that NLRP3 in renal tubular cells re-localizes from the cytosol to the mitochondria during hypoxia and binds to MAVS, which attenuates mtROS production and depolarization of the mitochondrial membrane potential under hypoxia (92). This evidence illustrates that the activated NLRP3 inflammasome re-localizes within the cell-driven mitochondrial dysfunction in cancer cells, resulting in metabolic reprogramming and cancer progression.

**Table 3:** Metabolism- and genome instability-associated cancer hallmarks.

Cancer type	Mechanism	Reference
***Deregulating cellular energetics- aerobic glycolysis***		
Breast	Obesity promotes the progression *via* the NLRC4 activation	(42)
Colitis-associated cancer	NLRP3 activation increases IL-1β in response to glycolysis	(86)
Lung	NALP3 ablation led to decreased glycolysis and enhanced mitochondrial respiration, may play a tumor-promoting role in NSCLC	(83)
NPC	Activation of glycolysis activates NLRP3 and finally changes the environment of malignant cells resulting in NPC-derived MDSC	(84)
***Genome instability***		
Lung	ATX transfection induces NLRP3 activation and DNA damages	(87)
Skin	NLRP3 is activated by UVB-induced DNA damage and releases inflammatory mediators	(88)

A causal mechanism by which obesity promotes the progression of breast cancer *via* the NLRP3 inflammasome activation has been recently described (42, 93). Wen et al. pointed out that IL-1β and NLRP3 inflammasome regulate lipid and carbohydrate metabolism. Enhanced NLRP3 inflammasome activation and processing of inactive pro-IL-1β to the active mature IL-1β are induced in response to administration of saturated fatty acids. Moreover, NLRP3 downstream IL-1β mRNA induction is required by glycolysis. Both glycolysis and HIF-1*α* expression are critical for Th17 cell differentiation (86) (**Table 3**). The AMP-activated protein kinase (AMPK), downregulated in cancer and obesity, has been proposed to mediate survival *via* its effect on cellular metabolism (94), through oncogenic progression of re-configuring/re-programming its metabolism towards glycolysis. AMPK inhibition blocks autophagy and increases mtROS production, a trigger signal for NLRP3 inflammasome activation (95).

### Genome Instability

Genomic instability is a characteristic of most cancers, resulting from mutations in DNA repair genes and drives cancer development (96). A recent study indicated that short-wave ultraviolet B (UVB) exposure activates the NLRP3 inflammasome and induces the secretion of IL-1β and IL-18 (97). In addition, a positive correlation has been found between UVB radiation intensity and NLRP3 gene expression (88). Knockdown of NLRP3 suppresses UVB-induced production of IL-1β and attenuates other inflammatory mediators, such as IL-1α, IL-6, TNF-α and PGE_2_. Inhibition of the DNA repair system by knockdown XPA, a major component of nucleotide excision repair, causes activation of NLRP3 inflammasome. Thus, Hasegawa et al. indicated that accumulating genomic damage due to loss of repair would result in NLRP3 activation (88). ATM-activated autotaxin (ATX) propagates inflammation and accumulates DNA double-strand breaks (DSBs) in epithelial cells. Zheng et al. demonstrated that ATX transfection induces NLRP3 activation and DNA damages. These results suggest that ATX is activated *via* stress-induced NLRP3-DSB-ataxia telangiectasia-mutated (ATM) response (87) (**Table 3**).

### Inducing Angiogenesis

To satisfy the highly demanding metabolic requirements, cancer switches on aberrant angiogenesis to help sustain expanding neoplastic growth. The role of NLRP3 in tumor-associated neo-vasculature and angiogenesis has been found to regulate angiogenesis in CRC, breast cancer, NSCLC (21, 53). Numerous studies have pointed out the angiogenic role of IL-1β (42, 46, 98). The circuitry of angiogenesis is proposed to be activated through numerous autocrine and paracrine actions. Carmi et al. indicated that IL-1β and vascular endothelial growth factor (VEGF) interact in an autoinduction circuit where these cytokines would result in the positive feedback of the other. The circuitry is proposed to be orchestrated between bone marrow-derived VEGF receptor 1/IL-1 receptor 11 expressed MDSC and tissue endothelial cells (ECs). Inhibition of IL-1β reduces tumor growth in melanoma by attenuating inflammation and promoting MDSC towards M1 macrophage, which subsequently reduces angiogenesis through neutralized VEGF; however, when tumors relapse, the expression of HIF-1*α* in a paracrine manner has been found to allow angiogenesis to rebound. These findings suggest that IL-1β inhibition acts as an effective anti-tumor therapy in targeting tumor angiogenesis (98) (**Table 4**). 

**Table 4:** Metastasis-associated cancer hallmarks.

Cancer type	Mechanism	Reference
***Inducing angiogenesis***		
Breast	TAMs take a center stage in promoting both tumor angiogenesis and metastatic spread	(22)
Breast	IL-1β and the chemotactic factor CCL2 activate CXCL12 promoting angiogenesis	(42)
Breast	Inactivation of NLRP3 decreases angiogenesis	(53)
Colon	NLRP3 inhibits angiogenesis	(21)
Melanoma	IL-1β induces VEGF to provide the inflammatory and pro-angiogenic TME	(98)
NSCLC	NLRP3 in GSDMD-deficient tumor cells inhibits EGFR/Akt signaling	(68)
Prostate	Mice deficient in IL-1β exhibits impaired tumor development and blood vessel growth	(99)
***Activating invasion and metastasis***		
Breast	Decreasing NLRP3 induced cell apoptosis and decreased cell migration	(54)
Endometrial	NLRP3 enhances proliferation, migration, and invasion	(55)
Gastric	NLRP3 promotes migration and invasion	(24)
Glioma	NLRP3 promotes migration and invasion	(56)
HCC	NLRP3 represses proliferation and metastasis	(21)
HNSC	Inhibition of the NLRP3 decreases tumor cell invasion and survival	(21)
***NF-κB and IL-1β in modulating EMT***		
Bladder	IL-1β promotes metastasis *via* inducing EMT or increases MMP activity	(24)
Breast	NLRP3 upregulated the expression of adhesion molecules on EC promotes metastasis	(23)
CRC	NLRP3 activated *via* TGF-*β*1 and TNF-α activates NF-*κ*B signaling that induces EMT	(100)
CRC	NLRP3 increases cell migration and invasion	(24, 42)
Lung	The negative feedback of downstream NLRP3 by miR-223 promotes invasion *via* activation of the NF-*κ*B signaling	(54)
Gastric	IL-1β activates NF-*κ*B that initiates JNK signaling causing invasion	(21)
OSCC	Silencing of NLRP3 decreases the migration and invasion and reduces EMT-related protein expression	(47)

More pathway details were revealed in the study by Voronov et al. where the authors established that IL-1 expression at TME enhances adhesion molecules on EC and facilitates tumor EMT and metastasis (101, 102). Although not regarded as the primary driver of angiogenesis, recombinant interleukin 1 (rIL-1) promotes the proliferation of EC and enhances the expression of adhesion molecules as well as inflammatory mediators and cytokines, which subsequently promote vascular permeability (103, 104). Thus, Voronov et al. aimed to assess the role of IL-1 as a driver of tumor angiogenesis and evaluate its potential as an anti-tumor agent, and by employing *IL-1β* knockout mice, the authors found that IL-1β is required for *in vivo* angiogenesis since *IL-1β* knockout mice failed to recruit blood vessel networks into Matrigel plugs with melanoma tumor cells. The incorporation of IL-1 receptor antagonists into these plugs also inhibited the ingrowth of blood vessel networks in wild-type mice. Similar to melanoma gel models, this phenomenon was consistently found in DA/3 mammary and prostate cancer cell models (99) (**Table 4**).

### Activating Invasion and Metastasis

The invasion and metastasis cascade not only induce in-and-out extravasation of tumor cells into and out of the lymphatic or vascular systems but additionally affect the dissemination and survival in the bloodstream and targeting of metastatic sites while evading immune surveillance. The role of NLRP3 in activating invasion and metastasis seems to be tissue- and context-dependent. Increased activation of the NLRP3 inflammasome promotes migration and invasion activities in gastric cancer cells (24). Consistently, knockdown of NLRP3 induces cell apoptosis in MCF-7 cells and decreases cell migration (54); nevertheless, in other cell-types, NLRP3 inflammasome may pharmacologically repress proliferation and metastasis of hepatic cell carcinoma (HCC) (21) (**Table 4**).

The role of NLRP3 in promoting invasion has been demonstrated with human endometrial cancer cell lines such as Ishikawa and HEC-1A cells, where knockdown of NLRP3 significantly reduces proliferation, clonogenicity, invasion and migration. In contrast, overexpression of NLRP3 enhances the activities of proliferation, migration and invasion as well as increasing caspase-1 activation and IL-1β secretion in human endometrial cancer cells (55). Liu et al. concluded that the upregulation of NLRP3 expression promotes the progression of endometrial cancer; therefore, NLPR3 inflammasome might be a new therapeutic target for endometrial cancer (55). Unanimously, inhibition of the NLRP3 inflammasome pathway has been suggested to be a promising approach for decreasing tumor cell invasion and survival in HNSC (21) and glioma (56) (**Table 4**).

The cascade of downstream pathways *via* activation of the NLRP3-mediated IL-1β axis has been found to activate NF-*κ*B that initiates JNK signaling causing proliferation and invasion in gastric cancer (21). On the other hand, despite the activation of the same downstream pathways, NF-*κ*B, NLRP3 is shown by numerous studies to be anti-oncogenic. MicroRNA-233-3p (miR-223-3p) has been found to directly target NLRP3, which results in diminishing the expression of NLRP3 and its downstream molecules, including ASC, IL-1β and IL-18, suggesting that miR-223-3p might be a dynamic control in determining inflammasome-mediated cell fate (54). Moreover, miR-223 has been demonstrated to increase proliferation, promote invasion and inhibit apoptosis in A549 cells *via* activation of the NF-*κ*B signaling pathway (105). Adding a layer of complexity into the intracellular crosstalk, NF-*κ*B signaling has also been proposed to act upstream of NLRP3 where NF-*κ*B is required for the upregulation of NLRP3 in transforming growth factor-beta 1 (TGF-β1)- or TNF-α-induced EMT in CRC (100) (**Table 4**). Whether the complexity of crosstalk remains to be unraveled or whether the role of NLRP3 is cell-specific remains to be elucidated. 

It is increasingly apparent that crosstalk between cancer cells and cells of the neoplastic stroma is involved in permitting seeding and successful colonization. The activation of NLRP3 inflammasome in breast CAF promotes tumor metastasis by upregulating the expression of adhesion molecules in EC and promoting the recruitment of MDSCs, suggesting that IL-1β secretion from activated mammary CAFs enhances the expression of adhesion molecules on tumor EC *in vivo*, thus facilitating cancer cell migration across the blood vessel endothelium (23). IL-1β has also been found to promote metastasis by inducing EMT or increasing matrix metalloproteinases (MMPs) activity in gastric adenocarcinoma, bladder cancer (24) and CRC (42). Moreover, the silencing of NLRP3 significantly decreases the migration and invasion in OSCC cells and reduces EMT-related protein expression. These results suggest that NLRP3 plays an important role in regulating the metastasis of OSCC cells (47) (**Table 4**).

## Discussion

The role of NLRP3 in tumorigenesis may be tissue- or cell-dependent. Conflicting roles have been described where NLRP3 exhibits a protective role against colon cancer and HCC, while NLRP3 also plays a carcinogenic role in gastric and prostate cancers (39, 106). The multitude of cellular impacts demands a comprehensive analysis of tissue-context response, dose-dependent response, and *in vivo* implications that require further evaluation. Would these complicate benefits in multiple metastatic tumor responses or high-risk patients with cancer susceptibilities? If the drug is to be spread systematically through the oral or intravenous route, what will the overall response be considering immune priming distance from the primary site?

NLRP3 using conditional knockout models and pharmacological activators or inhibitors is needed to decode the precise mechanisms of NLRP3 activity in cancer (39). Awad et al. found that the NLRP3 effector ASC protein plays a differential regulation of NF-*κ*B in different clinical scenarios. In metastatic melanoma, ASC encourages tumorigenesis while suppressing tumorigenesis in primary melanoma. The tissue-specific function of ASC is then confirmed *in vivo* when ASC acts as a tumor suppressor in keratinocytes and an oncoprotein in myeloid cells (48). Observing that inflammasome pathways serve dual opposing functions, Duan et al. proposed that NLRP3 activation could be enhanced in cells upon stimulated inflammation by LPS and coal tar pitch extract (CTPE) exposure compared to CTPE alone (107). Whether NLRP3 activation may result in proliferation or apoptosis of the cell would depend on the level of stress imposed, and the sensitivity of the cell to produce IL-1β requires elucidation. Would apoptosis be the result from overwhelming stress and pro-tumorigenic response as a consequence of less severe damage, repairable stress? An interesting question that arises would be whether the cell status predicts cell fate and its preposition towards apoptosis and senescence. 

While seemingly bizarre with unique cell-dependent phenotypes and crosstalk, NLRP3 at the setting seems to be organized and goal-dependent. Where many uncertainties are regulated and compelled to arrive at one sole cell fate, inhibiting NLRP3 activation with *S. muticum* extracts has been found to decrease angiogenesis, reduce cancer cell proliferation, and increase apoptosis in human breast cancer cells (53). NLRP3 enhances IL-1β, subsequently activating NF-*κ*B, and initiates JNK signaling to cause proliferation and invasion in gastric cancer (21). In lung cancer, it increases proliferation, promotes invasion and inhibits apoptosis (54). The knockdown of NLRP3 significantly reduces the proliferation, clonogenicity, invasion and migration in both Ishikawa and HEC-1A cells, while in contrast, NLRP3 overexpression enhances the proliferation, migration and invasion in both Ishikawa and HEC-1A cells and furthermore, increases caspase-1 activation and the release of IL-1β in endometrial cancer cells. YVAD-cmk, an inhibitor of caspase-1, decreases the proliferation ability.

Collectively, these results indicate that upregulated NLRP3 expression promotes the progression of endometrial cancer (55). The multifunctional role of NLRP3 in mutually conflicting pathways raises the question of what determines deterministic cell fate decisions in development and oncogenesis. And since inflammasome is a constitutively and physiologically expressed protein in maintaining homeostasis, would the target of NLRP3 inflammasome mitigate malignancy without harming normal neighboring tissue? Addressing these concerns, Bae et al. proposed that significant cell death was observed only when P2X7R and NLRP3 inflammasome were both inhibited by ATP and MCC950, a specific inhibitor of NLRP3 inflammasome (108), and further research into safety manipulation of NLRP3 inflammasome without enhancing significant dose-dependent side effects is required.

**Figure 2:**
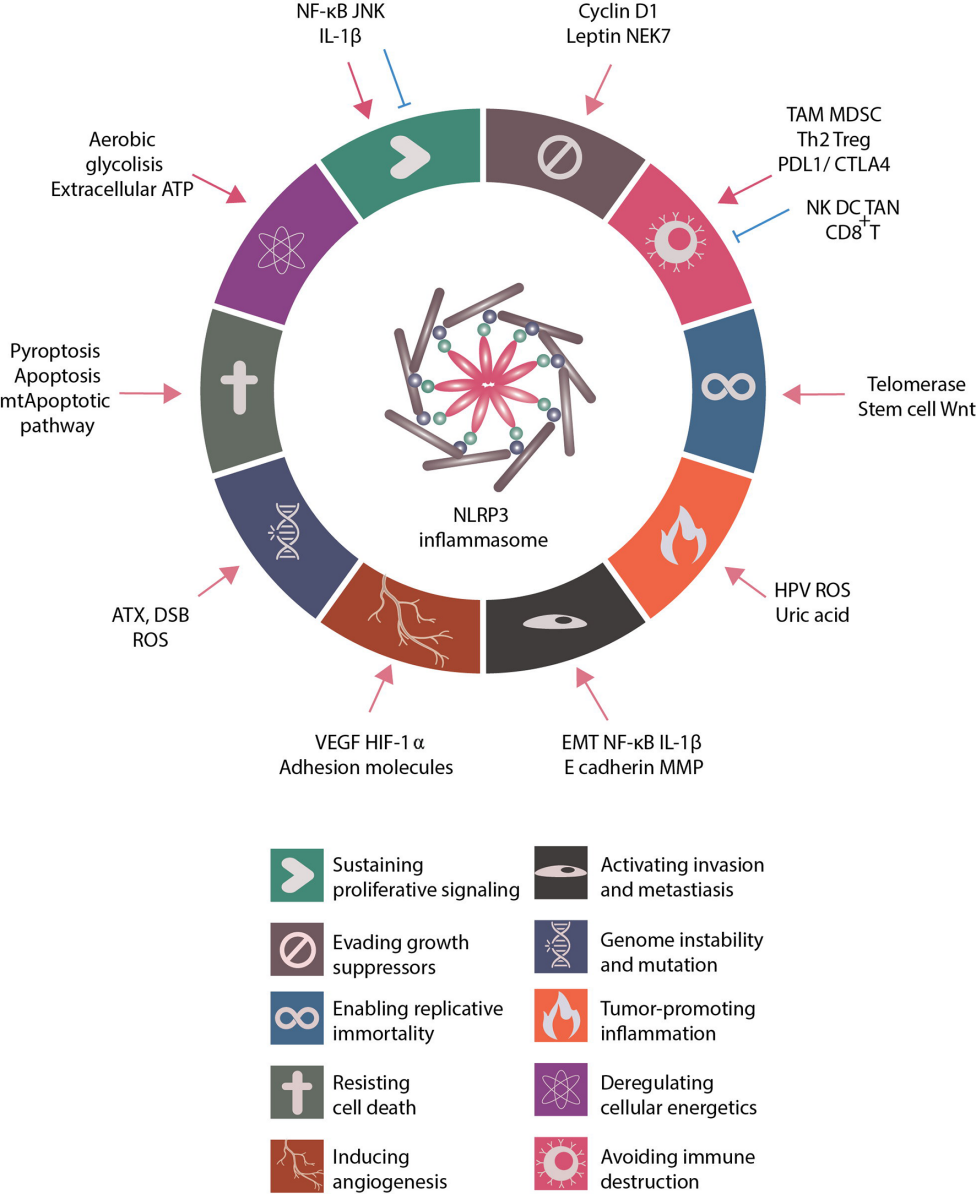
Role of NLRP3 inflammasome in regulating cancer hallmarks. Proposed by Dr. Douglas Hanahan and Dr. Robert A. Weinberg, the cancer hallmarks are universal abilities that cancer cells acquire for survival. These hallmarks include sustaining proliferative signaling, evading growth suppressors, resisting cell death, enabling replicative immortality, inducing angiogenesis, activating invasion and metastasis, deregulating cellular energetics, avoiding immune destruction, tumor-promoting inflammation and genome instability and mutation. The related literature was reviewed and the role of NLRP3 inflammasome in regulating cancer hallmarks summarized. Many NLRP3 inflammasome-related inducers and NLRP3 inflammasome-associated regulations modulate the activities of cancer hallmarks.

It is important not to overlook that EMT, invasion, survival, proliferation, angiogenesis and immune invasion-all traits are implicated in the process of invasion and metastasis (**Figure 2**). Hence, the distinct crosstalk highlighted in this review implies intertwined processes that are not independently activated and that act synergistically to allow tumor clonogenicity expansion and progression. Given the importance and multifaceted role of inflammasome/inflammation in cancer biology, the complexity of NLRP3 is just beginning to be decoded where inflammasome not only acts as a critical regulator in modulating tumor-associated inflammation but mediates essential roles in regulating cancer hallmarks as well. In response to a variety of stresses, mammalian cells activate the inflammasome for targeted pyroptosis; however, inflammasome has been associated with tumor-promoting effects, raising the interesting question of why inflammasome-mediated pyroptosis fails to outcompete the superior cell fate option in many dysregulated cellular contexts. What are the missing key drivers that will promote the major downstream event of caspase activation and subsequent pyroptosis in mediating tumor control? These recent developments provide new insights and therapeutic opportunities to manipulate inflammasome in augmenting their anti-tumor activity and achieving metastatic control for therapeutic opportunities.

## Author Contributions

T-YL, S-PH, and C-YL contributed to the conception and design of the review study. T-YL, M-CT, and WT wrote the first draft of the manuscript. T-YL, M-CT, WT, H-CY, S-CW, and C-YL wrote sections of the manuscript. T-YL, S-PH, and C-YL discussed and revised the content of the review article. All authors contributed to the article and approved the submitted version.

## Funding

This study was supported in part by grants from the Ministry of Science and Technology, Taiwan, R.O.C. (grant nos. MOST 108-2320-B-037-007, 109-2320-B-037-007-MY3, 109-2314-B-037-108-MY2, 108-2314-B-037-029, and 109-2314-B-037-106-MY3), Kaohsiung Medical University Research Center Grant (KMU-TC108A04), and Kaohsiung Medical University Hospital (grant no: KMUH105-5R42).

## Conflict of Interest

The authors declare that the research was conducted in the absence of any commercial or financial relationships that could be construed as a potential conflict of interest.
